# Effects of Green Tea Extract and (+)‐Catechin Hydrate on Sperm Quality and Seminal Plasma Antioxidant Activity: An In Vitro Study in Rams

**DOI:** 10.1002/vms3.71015

**Published:** 2026-06-02

**Authors:** Robab Nabhani, Saleh Tabatabaei Vakili, Khalil Mirzadeh

**Affiliations:** ^1^ Department of Animal Science Faculty of Animal Science and Food Technology Agricultural Sciences and Natural Resources University of Khuzestan Mollasani Iran

**Keywords:** catechin, chilled semen, green tea, malondialdehyde, sperm quality

## Abstract

**Background:**

Antioxidants have gained significant attention for improving sperm parameters. Green tea and catechin, known for their potent antioxidant properties, are particularly interesting in this context.

**Objective:**

This study investigated the in vitro effects of green tea extract (GTE) and (+)‐catechin on semen quality parameters of Arabi rams during chilled storage.

**Methods:**

The pooled and diluted semen was divided and supplemented with GTE and (+)‐catechin (50, 100, 150 and 200 µg/mL levels). The control group did not receive any supplements. Sperm quality and semen pH were evaluated at 1, 24, 48 and 72 h after storage. Seminal plasma MDA level was measured at 72 h of semen storage.

**Results:**

Supplementation with GTE and (+)‐catechin improved sperm motility more than control (*p* < 0.05). Specifically, GTE at 50 and 100 µg/mL enhanced motility, while higher concentrations were detrimental. Catechin at 600 and 800 µg/mL maintained motility better than the control at 72 h. Sperm viability and membrane integrity were significantly improved at 50 and 100 µg/mL GTE and all catechin concentrations (*p* < 0.05) compared to the control. However, higher GTE concentrations (150‐200 µg/mL) decreased sperm viability and membrane integrity at 72 h. Additionally, morphological abnormalities were reduced by 100 µg/mL GTE and the higher catechin concentrations in comparison to the control. Seminal plasma pH varied over the 72‐h storage period, but no significant differences were observed between the experimental and control. MDA levels were significantly lower (*p* < 0.05) in the GTE (50–200 µg/mL) and catechin (200–800 µg/mL) treatment groups, compared to the control at 72 h.

**Conclusions:**

GTE and catechin enhance semen parameters during chilled storage, with optimal effects at specific concentrations. High levels of GTE may negatively impact sperm quality, underscoring the importance of precise dosing in semen preservation.

## Graphical abstract

This study investigated the in vitro effects of green tea extract (GTE) and (+)‐catechin on semen quality parameters of Arabi rams during chilled storage. The results showed that GTE and catechin enhance semen parameters during chilled storage, with optimal effects at specific concentrations. High levels of GTE may negatively impact sperm quality, underscoring the importance of precise dosing in semen preservation.

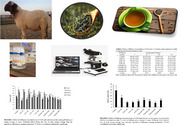

## 1 Introduction

The lipid composition of sheep sperm membranes differs significantly from that of somatic cells, being rich in polyunsaturated fatty acids (PUFAs), which make sperm more vulnerable to oxidative damage from reactive oxygen species (ROS) during storage (Wang et al. 2022). ROS plays a critical role in both reproductive physiology and pathology, with its effects depending on factors like concentration, source, and exposure time (Qamar et al. 2023). Oxidative stress, which results from an imbalance between ROS production and antioxidant defences, has been linked to defective sperm function (Wang et al. 2025). Excessive ROS can damage sperm membranes, decrease fertility and reduce semen quality (Dabagh et al. 2023).

Antioxidants counteract oxidative damage by reducing the rate of oxidation (Agarwal and Sekhon 2010). The total antioxidant capacity of seminal plasma arises from both enzymatic and nonenzymatic properties. Low levels of this capacity are associated with male infertility (Słowińska et al. 2013). Moreover, the antioxidant content in sperm is limited and declines further during semen dilution and storage. Supplementing extenders with antioxidants during semen preservation can therefore enhance sperm quality (Riesco et al. 2021). For example, malondialdehyde (MDA), a byproduct of lipid peroxidation, is elevated in semen when lipid peroxidation occurs, which negatively impacts sperm fertility (Ghiasi Ghalehkandi 2014).

In the past, artificial antioxidants were used to protect sperm from oxidative stress. However, concerns over cytotoxicity have led researchers to explore natural antioxidants as safer alternatives (Malo et al. 2011). Plant extracts, rich in antioxidants such as phenolics, saponins, alkaloids, vitamins and minerals, help reduce oxidative stress, balance free radicals and decrease lipid peroxidation in sperm (Ros‐Santaella and Pintus 2021).

Green tea (*Camellia sinensis*), a plant from the Theaceae family, is a rich source of natural antioxidants, including polyphenols, methylxanthines, vitamins (A, C, E and B), and minerals such as chromium, magnesium, selenium, and zinc (Biasibetti et al. 2013). The composition of green tea also includes lipids, proteins, fibres, and significant amounts of polyphenols (Setumo et al. 2022; Murokore et al. 2023). The manufacturing process, which involves drying, steaming, or roasting the leaves, preserves these antioxidant compounds by deactivating oxidative enzymes (Choi et al. 2016).

The catechins in green tea, particularly (+)‐catechin, represent a major group of antioxidants, accounting for 25%–35% of the dry weight. Other key catechins in green tea include (−)‐epicatechin (EC), (−)‐epigallocatechin (EGC), (−)‐epicatechin gallate (ECG), and (−)‐epigallocatechin gallate (EGCG), with EGCG being the most abundant (Forester and Lambert 2011; Nain et al. 2022). (+)‐Catechin is a potent antioxidant with potential health benefits, including supporting cardiovascular health and preventing chronic diseases (Patanè et al. 2023; Rai et al. 2020).

Previous studies on the effects of green tea on male reproductive health have shown inconsistent results (Opuwari and Monsees 2020). However, research indicates that green tea extract (GTE) can inhibit peroxidation of the sperm plasma membrane and maintain the viability of frozen semen (Khan et al. 2017). Moreover, adding tea extract to semen extenders has been shown to improve sperm viability in a dose‐dependent manner (Dias et al. 2014).

Given the therapeutic potential of green tea and its active compounds, coupled with the antioxidant properties of catechins, the present study aims to evaluate the effects of catechin and GTE on sperm quality and lipid peroxidation in Arabi rams.

## 2 Materials And Methods

### 2.1 Research Location and Animals

The present study was conducted from January to March, during the non‐breeding season, at the research farm of the Agricultural Sciences and Natural Resources University of Khuzestan, located in Khuzestan province, Iran. A total of 10 Arabi rams (Ovis aries), aged 3 ± 0.5 years, were used for this experiment. The rams were sourced from the university's breeding programme and transported to the research facility. The animals were housed indoors as a group, with free access to water and were fed a diet formulated in accordance with the National Research Council (NRC, 2016) guidelines. The diet consisted of alfalfa (17%), barley (53%), wheat bran (13%), soybean meal (3%), oyster powder (0.5%) and wheat straw (12%), which provided a crude protein content of 12.88% and metabolisable energy of 2.53 Mcal/kg.

### 2.2 Semen Collection and Preparation

Semen were collected using an electro‐ejaculator device from each ram twice a week for 6 weeks. Samples were immediately transferred to the laboratory and incubated at 37°C in a water bath. Only ejaculates with a volume of 1–2 mL, a minimum spermatozoa concentration of 2 × 10^9^/mL, and greater than 80% sperm motility were used. The semen samples were pooled to reduce errors due to individual differences and diluted in Tris base extender (Tris: 3.63 g, fructose: 0.5 g, citric acid: 1.99 g, egg yolk: 14%, glycerol: 5%, penicillin: 100,000 IU, streptomycin: 100 mg, distilled water: 100 mL) in a ratio of 1:10.

### 2.3 Semen Processing and Experimental Groups

The pooled and diluted semen was divided into nine equal aliquots and supplemented with different concentrations of GTE and (+)‐catechin (Sigma‐Aldrich). The control group did not receive any supplements, while the other experimental groups received varying levels of GTE (50, 100, 150 and 200 µg/mL) and catechin (200, 400, 600 and 800 µg/mL). The main chemical components of GTE detected by GC‐MS include Polyphenols, volatile compounds, caffeine, lipids and fatty acids, citric acid, terpenes and tannins.

### 2.4 Assessment of Sperm Quality Parameters

The treated semen samples were stored at 4°C in a refrigerator, and sperm quality parameters were examined at 1, 24, 48 and 72 h after storage. Sperm total motility and progressive motility were estimated using computer‐assisted sperm motility analysis (CASA, video test‐sperm 2.1, St. Petersburg, Russia). Five microscopic fields were analysed for each semen sample (Santos et al. 2019). Sperm plasma membrane integrity was evaluated using a hypotonic swelling test (HOST). A 5 µL semen sample was mixed with 50 µL hypo‐osmotic solution (1 g fructose and 0.735 g sodium citrate dissolved in 100 mL distilled water) and incubated at 37°C for 30 min. The percentage of swollen and coiled tails in 200 sperm was counted using a 400x phase contrast microscope and considered indicative of normal plasma membrane integrity (Zubair et al. 2014; Wang et al. 2022). Sperm viability was evaluated using the eosin‐nigrosin staining method. A small drop (10 µL) of semen was placed on a pre‐warmed slide and mixed with a larger drop (20 µL) of the stain (10 g nigrosin, 1.7 g eosin and 2.9 g sodium citrate dissolved in 100 mL distilled water). After drying, viability was assessed by counting 200 cells by microscope at 1000× magnification using immersion oil. Spermatozoa with unstained sperm heads were considered viable, while sperm with stained or partially stained heads were counted as dead (Amini et al. 2015).

Morphological sperm abnormalities were determined by examining semen smears stained with eosin‐nigrosin on a glass slide. A total of 200 spermatozoa were counted on each slide at 1000× magnification using immersion oil, and the percentage of morphologically abnormal sperms in the head, mid‐piece and tail was estimated (Azubuike et al. 2017).

### 2.5 Assessement of MDA in Seminal Plasma

The level of MDA in seminal plasma was measured at 72 h of semen storage as an indicator of lipid peroxidation using the thiobarbituric acid (TBA) test. One mL of the seminal plasma was mixed with one mL of cold 20% (w/v) TBA. To inhibit lipid oxidation, one mL of butylated hydroxytoluene (2% BHT solution in ethanol) and one mL of ethylenediaminetetraacetic acid (EDTA) were added to the sample before trichloracetic acid (TCA) precipitation. The precipitate was pelleted by centrifuging (1200 g for 15 min), and one mL of the supernatant was incubated with one mL of 0.67% (w/v) TBA in a boiling water bath at 100°C for 10 min. After cooling, the absorbance was determined using a spectrophotometer at 530–540 nm, and the MDA content was expressed as nmol/mL (Amini et al. 2015; Daghigh Kia and Vatankhah 2019).

### 2.6 Statistical Analysis

The study included nine experimental groups, consisting of a control group and eight treatment groups, where GTE was supplemented at concentrations of 50, 100, 150 and 200 µg/mL, and (+)‐catechin at concentrations of 200, 400, 600 and 800 µg/mL. Semen samples were stored under chilled conditions, and sperm quality parameters were evaluated at 1, 24, 48 and 72 h after storage. The data were analysed using one‐way ANOVA to evaluate differences in sperm quality and seminal plasma MDA level between the treatment groups at each time point. Post hoc comparisons were conducted using Duncan's multiple range test to determine which specific treatment groups differed. All statistical analyses were performed using SPSS software (version 22.0, Chicago, Illinois), with a significance level of *p* < 0.05 set for all tests.

## 3 Results

### 3.1 Sperm Motility

The results presented in Table 1 show that GTE and catechin at different levels have a significant effect (*p* < 0.05) on sperm motility during semen storage in chilled conditions. After 1, 24, 48 and 72 h of semen storage, the applied levels of catechin performed better in maintaining overall sperm motility compared to the control and GTE (*p* < 0.05). As the semen storage period increased, 50 and 100 µg/mL of GTE improved the percentage of sperm motility, while higher levels of the extract had a detrimental effect on sperm motility.

**TABLE 1:** Effect of different concentrations of GTE and (+)‐catechin on the total motility (%) of spermatozoa during chilled semen storage in rams.

Treatments (µg/mL)	Storage period (hours)			
	1	24	48	72
Control	76.67 ±5.27^bc^	60.00 ± 5.02^de^	46.67 ± 4.22^c^	35.00 ± 3.16^f^
GTE 50	84.17 ±5.68^ab^	73.33 ± 6.15^cd^	64.17 ± 4.55^b^	50.83 ± 2.01^e^
GTE 100	89.16 ± 4.73^ab^	75.83 ± 2.39^bc^	66.67 ± 3.80^b^	55.83 ± 3.00^de^
GTE 150	69.17 ± 6.51^cd^	60.84 ± 5.83^de^	46.66 ± 4.94^c^	33.33 ± 5.42^f^
GTE 200	61.66 ± 5.58^d^	53.33 ± 5.27^e^	44.17 ± 3.75^c^	28.34 ± 3.33^f^
Catechin 200	92.50 ± 2.14^a^	86.00 ± 3.67^abc^	77.50 ± 4.23^a^	62.50 ± 2.81^cd^
Catechin 400	93.33 ± 2.11^a^	87.50 ± 3.59^ab^	80.83 ± 3.27^a^	66.67 ± 2.79^bc^
Catechin 600	94.17 ± 2.39^a^	90.00 ± 2.89^a^	86.67 ± 2.79^a^	74.17 ± 2.71^ab^
Catechin 800	95.83 ± 2.01^a^	92.50 ± 1.44^a^	89.17 ± 1.54^a^	78.33 ± 1.05^a^
*p*‐value	0.011	0.015	0.021	0.017

*Note*: Data presented as Mean ± SE, Duncan test. Means with a different superscript in the same column differ significantly. *p* < 0.05 is considered statistically significant.

The results of this study indicated that GTE and catechin improved the progressive motility of spermatozoa compared to the control group in a dose‐dependent manner (*p* < 0.05). During different periods of semen storage, the percentage of sperm progressive motility initially increased with increasing GTE concentration compared to the control but decreased when the concentration of the extract reached 150 and 200 µg/mL (*p* < 0.05). Conversely, with the increase in catechin concentration, sperm progressive motility increased, and after 72 h of semen storage, the highest (*p* < 0.05) progressive motility was observed at 600 and 800 µg/mL of catechin (Table 2).

**TABLE 2:** Effect of different concentrations of GTE and (+)‐catechin on the progressive motility (%) of spermatozoa during chilled semen storage in rams.

Treatments (µg/mL)	Storage period (hours)			
	1	24	48	72
Control	71.00 ± 4.69^cd^	52.50 ± 5.26^d^	37.67 ± 4.01^d^	28.00 ± 3.26^f^
GTE 50	75.83 ± 4.67^bcd^	65.17 ± 5.71^bc^	55.67 ± 3.77^c^	43.02 ± 3.35^e^
GTE 100	81.67 ± 4.94^abc^	66.83 ± 2.40^b^	57.83 ± 3.22^c^	46.83 ± 2.97^de^
GTE 150	62.00 ± 5.03^de^	53.83 ± 6.05^cd^	39.33 ± 4.40^d^	26.67 ± 4.96^f^
GTE 200	55.33 ± 5.36^e^	47.17 ± 3.36^d^	36.17 ± 4.28^d^	19.17 ± 2.44^f^
Catechin 200	87.00 ± 3.32^ab^	78.17 ± 3.96^ab^	68.50 ± 3.55^b^	53.83 ± 2.81^cd^
Catechin 400	85.83 ± 3.00^ab^	77.83 ± 3.95^ab^	72.83 ± 3.69^ab^	57.80 ± 2.30^bc^
Catechin 600	90.00 ± 2.58^a^	84.17 ± 2.71^a^	79.17 ± 2.57^ab^	64.39 ± 2.79^ab^
Catechin 800	92.50 ± 1.87^a^	85.33 ± 2.60^a^	82.67 ± 1.76^a^	71.00 ± 1.59^a^
*p*‐value	0.018	0.014	0.010	0.026

*Note*: Data presented as Mean ± SE, Duncan test. Means with a different superscript in the same column differ significantly. *p* < 0.05 is considered statistically significant.

### 3.2 Sperm Viability, Plasma Membrane Integrity and Morphology

The effects of different levels of GTE and catechin on the viability, plasma membrane integrity and morphology of spermatozoa are presented in Tables 3, 4, 5. With the increase in the semen storage period, the concentrations of 50 and 100 µg/mL of GTE and all levels of catechin caused a significant improvement in the viability and plasma membrane integrity of sperm compared to the control (*p* < 0.05). However, 150 and 200 µg/mL GTE had no protective effect on sperm viability (Table 3). Based on the results, the higher doses of GTE not only failed to improve sperm plasma membrane integrity but also, at 72 h of semen storage, the highest level of the extract (200 µg/mL) significantly decreased this sperm parameter compared to the control group (Table 4). Regarding sperm morphology, at one and 24 h after semen storage, the percentage of sperm morphological abnormalities was not affected by the experimental treatments. However, at 48 and 72 h after semen storage in liquid condition, 100 µg/mL GTE and 600 and 800 µg/mL catechin significantly (*p* < 0.05) decreased the percentage of sperm morphological abnormalities compared to the control (Table 5).

**TABLE 3:** Effect of different concentrations of GTE and (+)‐Catechin on the spermatozoa viability (%) during chilled semen storage in rams.

Treatments (µg/mL)	Storage period (hours)			
	1	24	48	72
Control	80.83 ± 5.23^bc^	63.33 ± 4.22^c^	49.83 ± 4.81^e^	41.17 ± 4.66^f^
GTE 50	87.50 ± 5.28^ab^	78.40 ± 4.59^b^	69.50 ± 4.30^d^	54.67 ± 2.47^e^
GTE 100	90.10 ± 5.28^ab^	79.17 ± 2.03^b^	73.00 ± 3.21^cd^	59.50 ± 2.54^de^
GTE 150	70.83 ± 5.97^cd^	62.50 ± 5.59^c^	52.67 ± 4.18^e^	41.67 ± 4.22^f^
GTE 200	64.17 ± 4.73^d^	55.00 ± 3.65^c^	49.50 ± 4.44^e^	31.33 ± 4.05^g^
Catechin 200	95.83 ± 2.01^a^	91.67 ± 2.47^a^	82.67 ± 3.90^bc^	65.83 ± 2.55^cd^
Catechin 400	97.50 ± 1.71^a^	91.00 ± 1.93^a^	85.17 ± 2.18^ab^	70.17 ± 3.38^bc^
Catechin 600	98.33 ± 1.67^a^	93.33 ± 2.79^a^	90.33 ± 2.82^ab^	76.83 ± 2.48^ab^
Catechin 800	97.50 ± 1.59^a^	95.00 ± 1.84^a^	94.17 ± 0.83^a^	82.50 ± 1.71^a^
*p*‐value	0.021	0.010	0.030	0.014

*Note*: Data presented as Mean ± SE, Duncan test. Means with a different superscript in the same column differ significantly. *p* < 0.05 is considered statistically significant.

**TABLE 4:** Effect of different concentrations of GTE and (+)‐catechin on the spermatozoa membrane integrity (%) during chilled semen storage in rams.

Treatments (µg/mL)	Storage period (hours)			
	1	24	48	72
Control	75.80 ± 5.97^bc^	58.27 ± 6.01^c^	46.67 ± 4.22^c^	35.00 ± 3.18^d^
GTE 50	80.73 ± 5.69^ab^	70.00 ± 5.32^b^	63.33 ± 4.78^b^	49.90 ± 1.83^c^
GTE 100	83.33 ± 5.58^ab^	74.20 ± 2.71^b^	65.03 ± 2.89^b^	55.83 ± 3.01^bc^
GTE 150	65.35 ± 5.66^cd^	57.40 ± 4.96^c^	45.10 ± 4.83^c^	30.78 ± 4.73^d^
GTE 200	59.23 ± 4.30^d^	52.50 ± 4.23^c^	44.17 ± 3.75^c^	21.67 ± 3.57^e^
Catechin 200	88.34 ± 3.04^ab^	86.83 ± 2.45^a^	73.40 ± 3.07^ab^	60.76 ± 1.90^b^
Catechin 400	90.00 ± 1.29^a^	86.66 ± 2.79^a^	79.17 ± 3.35^a^	64.25 ± 2.49^ab^
Catechin 600	90.03 ± 1.83^a^	87.50 ± 1.12^a^	82.53 ± 2.14^a^	70.67 ± 2.01^a^
Catechin 800	91.67 ± 1.67^a^	88.34 ± 2.79^a^	84.16 ± 2.39^a^	72.44 ± 2.14^a^
*p*‐value	0.018	0.024	0.039	0.035

*Note*: Data presented as Mean ± SE, Duncan test. Means with a different superscript in the same column differ significantly. *p* < 0.05 is considered statistically significant.

**TABLE 5:** Effect of different concentrations of GTE and catechin on the spermatozoa morphological abnormalities (%) during chilled semen storage in rams.

Treatments (µg/mL)	Storage period (hours)			
	1	24	48	72
Control	4.33 ± 0.49	7.17 ± 0.80	11.67 ± 0.84^ab^	15.00 ± 1.83^abc^
GTE 50	3.67 ± 0.42	7.33 ± 0.83	9.33 ± 1.94^bc^	14.17 ± 1.54^bc^
GTE 100	4.17 ± 0.54	6.50 ± 0.92	7.83 ± 1.01^c^	12.50 ± 1.12^cd^
GTE 150	4.42 ± 0.49	6.83 ± 0.79	12.33 ± 1.25^a^	16.67 ± 0.84^ab^
GTE 200	4.50 ± 0.51	7.00 ± 0.82	12.00 ± 0.98^ab^	18.33 ± 1.67^a^
Catechin 200	3.66 ± 0.42	6.52 ± 0.50	9.83 ± 0.54^abc^	11.55 ± 0.84^cd^
Catechin 400	3.83 ± 0.40	6.60 ± 0.75	9.90 ± 0.90^abc^	11.17 ± 0.95^cd^
Catechin 600	3.72 ± 0.31	7.17 ± 0.31	8.33 ± 0.22^c^	10.03 ± 1.13^d^
Catechin 800	3.70 ± 0.39	6.67 ± 0.67	8.67 ± 0.45^c^	9.53 ± 0.72^d^
*p*‐value	0.52	0.22	0.003	0.008

*Note*: Data presented as Mean ± SE, Duncan test. Means with a different superscript in the same column differ significantly. *p* < 0.05 is considered statistically significant.

### 3.3 Seminal pH and MDA Level Analysis

**FIGURE 1:**
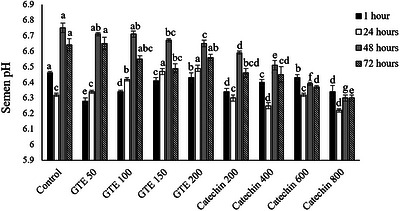
Effect of different concentrations of GTE and catechin on the semen pH during chilled semen storage in rams. Different letters above the bars in each semen storage time indicate significant differences among treatments. *p* < 0.05 is considered statistically significant.

**FIGURE 2:**
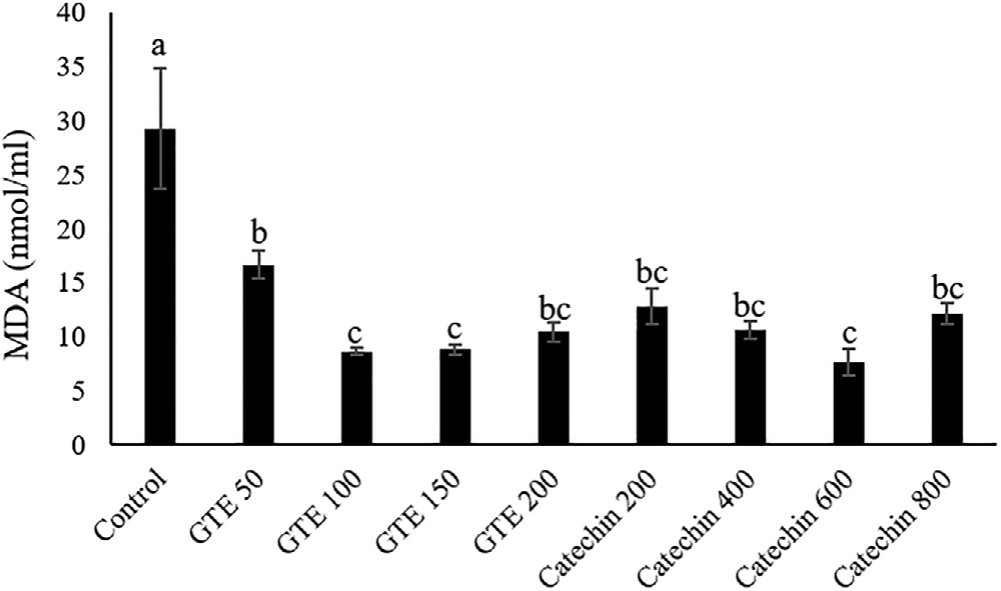
Effect of different concentrations of GTE and catechin on the semen malondialdehyde (MDA) concentration after 72 h of chilled semen storage in rams. Different letters above the bars in each semen storage time indicate significant differences among treatments. *p* < 0.05 is considered statistically significant.

The effects of different levels of GTE and catechin on seminal pH and MDA concentration are depicted in Figures 1 and 2. As the semen storage time increased, there were notable changes in semen pH across the experimental treatments. One hour after semen storage, the highest and lowest semen pHs were associated with the control and 50 µg/mL GTE, respectively. At 24 h, the highest pH of semen was observed in treatments containing GTE levels of 150 and 200 µg/mL. Subsequently, with the duration of semen storage reaching 48 and 72 h, the control group exhibited the highest pH, while catechin demonstrated lower semen pH in a dose‐dependent manner (*p* < 0.05) (Figure 1). Based on the findings, the addition of 50–200 µg/mL of GTE and 200–800 µg/mL of catechin to the diluent and storing the treated semen samples in chilled condition for 72 h resulted in a significant (*p* < 0.05) dose‐dependent decrease in MDA concentration compared to the control group (Figure 2).

## 4 Discussion

This study aimed to evaluate the effects of GTE and (+)‐catechin on semen quality parameters, including sperm motility, viability, plasma membrane integrity, morphology and oxidative stress, during chilled semen storage. Our findings indicate that both GTE and catechin significantly improve sperm quality in a dose‐dependent manner. These results provide insight into the potential use of these natural antioxidants in semen preservation and reproductive biotechnology.

The maintenance of sperm motility during semen storage is a critical factor for successful fertilisation, particularly in artificial insemination (AI). In our study, sperm motility improved significantly in GTE‐ and catechin‐treated groups compared to the control, with the highest motility observed at 50–100 µg/mL of GTE and 600–800 µg/mL of catechin. These results align with previous studies that reported positive effects of catechins on sperm motility. For example, Silva et al. (2019) demonstrated that GTE supplementation at 100 µg/mL improved sperm motility in goats, similar to our findings at the same concentration in rams. Also, adding GTE to human sperm freezing media at a lower concentration protected spermatozoa from the detrimental effects of cryopreservation, improving motility and DNA integrity (Alqawasmeh et al. 2021). Other studies also support the beneficial effects of GTE on sperm motility in dogs and roosters (Wittayarat et al. 2013; Hashemian and Mehri 2018). Contrarily, Baláži et al. (2022) reported that rabbits fed with a lower dose of green tea had decreased sperm motility and progressive motility, with a tendency for decreased progressive motility in the higher dose group. These differences may be due to the varying sensitivities or responsibilities of semen to grean tea across different experiments.

In our study, catechin concentrations (200–800 µg/mL) were effective in maintaining progressive motility, which is consistent with the findings of Keramati et al. (2023), who reported that catechins improved sperm motility in diabetic rats at comparable doses. However, our study also found that higher concentrations of GTE (150 and 200 µg/mL) decreased sperm motility, suggesting that excessive doses of GTE may have detrimental effects. This finding is in agreement with Roychoudhury et al. (2017), who noted that higher concentrations of green tea catechins could disrupt sperm motility due to an imbalance in antioxidant levels. In contrast, higher concentrations of catechin (600 and 800 µg/mL) maintained progressive motility, emphasising the importance of optimising dosage when using these compounds. This finding is in agreement with the study by Boosorn et al. (2010), which showed that catechin supplementation (50 and 25 µmol/L) improved sperm motility in pigs. The discrepancy could be due to species‐specific differences in sperm sensitivity to antioxidants or variations in the concentration of GTE and catechins used.

The protective effects of GTE and catechin on sperm viability and membrane integrity are well‐documented, and our study further supports these findings. Both GTE (50–100 µg/mL) and catechin (200–800 µg/mL) significantly enhanced sperm viability and plasma membrane integrity compared to the control. These results are in line with studies by Rai et al. (2020), who found that catechins improved sperm viability and motility without causing toxicity to the male reproductive system in rats. Moreover, Mustofa et al. (2021) observed that GTE supplementation (0.1 mg/100 mL) improved sperm viability in Kacang buck semen post‐thawing, similar to the improvements in sperm viability we observed with GTE. However, in our study, higher concentrations of GTE (150 and 200 µg/mL) failed to protect sperm viability, and at 200 µg/mL, it significantly reduced plasma membrane integrity after 72 h. This suggests that GTE may have a concentration threshold beyond which its beneficial effects are diminished. In one study, black tea extract (BTE) significantly improved sperm functions such as vitality and DNA fragmentation, attributed to its antioxidant properties, though there was no significant change in sperm motility (Setumo et al. 2023).

Similar to our study, Rahman et al. (2018) emphasised the importance of lower doses of polyphenols to reduce oxidative stress and improve fertility in both humans and animals. Moreover, Prastiya et al. (2023) observed that lower concentrations of GTE (0.15 mg/100 mL) enhanced sperm quality in bulls, which aligns with our findings that lower doses of GTE and catechin led to better sperm motility and membrane integrity. In contrast, Bucci et al. (2018) observed that different concentrations of EGCG did not improve the quality of frozen dog sperm after thawing.

Our results showed that the percentage of sperm abnormalities remained unaffected at 1 and 24 h, but significant improvements in sperm morphology were observed at 48 and 72 h following treatment with 100 µg/mL GTE and 600–800 µg/mL catechin. This is consistent with the findings of Kara et al. (2015), who reported that adding catechin to the breeder quail diet increased fertility and hatchability by improving sperm morphology. Similarly, Cimini et al. (2023) observed that catechins enhanced sperm fertilising ability in swine without negatively affecting sperm morphology, which aligns with our results showing reduced morphological abnormalities after prolonged storage. These findings support the notion that catechins may protect sperm structural integrity by reducing oxidative damage, as oxidative stress is known to cause sperm morphological abnormalities (Tvrda et al. 2019). On the other hand, the lack of improvement in sperm morphology at early time points (1 and 24 h) is likely due to the relatively short duration of exposure to the treatments, suggesting that the protective effects of GTE and catechin become more evident after prolonged semen storage.

Oxidative stress plays a critical role in semen quality degradation, and we observed that GTE and catechin supplementation resulted in a dose‐dependent decrease in MDA levels, a marker of lipid peroxidation. This decrease in MDA supports the antioxidant effects of GTE and catechins, which is consistent with previous studies. For instance, Inanç et al. (2019) reported that catechin supplementation in bull semen reduced oxidative stress and lipid peroxidation, enhancing semen quality. Similarly, Mustofa et al. (2021) found that GTE supplementation reduced MDA levels and improved post‐thaw sperm motility in Kacang buck. Oxidative stress reduction is crucial, as elevated ROS levels are known to impair sperm function by damaging cellular membranes, DNA, and proteins (Kaltsas 2023). Polyphenols might interact with components of the spermatozoa and would have decreased the lipid peroxidation induced by free radicals (Zhang et al. 2020). The positive effect of GTE may also stem from its metal and vitamin contents, which play critical roles in cell health by preventing oxidative stress induced by free radicals (Rai‐nishant et al. 2012). In rats treated with nonylphenols, as a suppressor of oxidative defence in living organisms, including the reproductive system, GTE consumption protected the testis structure and sperm parameters from nonylphenol toxication (Azizi and Mehranjani 2019).

The positive effects of GTE and catechin on sperm motility, viability and morphology, along with their ability to reduce oxidative stress, highlight their potential as additives in semen extenders for improving semen preservation. However, our study also emphasises the importance of dose optimisation. While lower concentrations of GTE and catechin improved sperm quality, higher concentrations were detrimental, particularly with GTE. This finding suggests that the use of antioxidants in semen extenders must be carefully calibrated to avoid negative effects.

## 5 Conclusion

The findings of this study indicate that the application of different concentrations of GTE and catechin positively influenced semen quality parameters across all time points. Specifically, GTE and catechin enhanced sperm motility, viability, concentration, morphology and membrane integrity. Significant improvements in motility were observed primarily at 24 and 48 h of storage, with higher concentrations of GTE and catechin providing the best results. Viability showed substantial enhancement at moderate‐to‐high concentrations, peaking at 48 h. Sperm concentration was notably higher in treatments with higher catechin and GTE concentrations, particularly at 72 h. Morphological improvements were evident at both 48 and 72 h, especially in treatments containing 5% GTE and moderate catechin levels. Membrane integrity was most significantly improved at 1 and 24 h, with moderate GTE and catechin concentrations yielding the best outcomes. These results suggest that moderate‐to‐high concentrations of GTE and catechin effectively maintain or improve semen quality, particularly within the first 48 h of storage.

## Author Contributions

**Robab Nabhani**: data curation, investigation, writing – original draft. **Saleh Tabatabaei Vakili**: methodology, formal analysis, supervision, writing – review and editing. **Khalil Mirzadeh**: supervision.

## Ethics Statement

The authors confirm that the ethical policies of the journal, as noted on the journal's author guidelines page, have been adhered to and the appropriate ethical review committee approval has been received. The US National Research Council's guidelines for the Care and Use of Laboratory Animals were followed.

## Conflicts of Interest

The authors declare no conflicts of interest.

## Funding

The authors have nothing to report.

## Data Availability

The data that support the findings of this study are available on request from the corresponding author.
